# Comparison of embedded and added motor imagery training in patients after stroke: study protocol of a randomised controlled pilot trial using a mixed methods approach

**DOI:** 10.1186/1745-6215-10-97

**Published:** 2009-10-22

**Authors:** Corina Schuster, Jenny Butler, Brian Andrews, Udo Kischka, Thierry Ettlin

**Affiliations:** 1 Reha Rheinfelden, Rheinfelden, Switzerland; 2 School of Health and Social Care, Oxford Brookes University, Oxford, UK; 3 School of Technology, Oxford Brookes University, Oxford, UK; 4 Oxford Centre for Enablement, Oxford, UK; 5 Department of Neurology, Medical faculty, University of Basel, Basel, Switzerland; 6 Department of Education, University of Applied Science Northwestern Switzerland, Basel, Switzerland

## Abstract

**Background:**

Two different approaches have been adopted when applying motor imagery (MI) to stroke patients. MI can be conducted either added to conventional physiotherapy or integrated within therapy sessions. The proposed study aims to compare the efficacy of embedded MI to an added MI intervention. Evidence from pilot studies reported in the literature suggests that both approaches can improve performance of a complex motor skill involving whole body movements, however, it remains to be demonstrated, which is the more effective one.

**Methods/Design:**

A single blinded, randomised controlled trial (RCT) with a pre-post intervention design will be carried out. The study design includes two experimental groups and a control group (CG). Both experimental groups (EG1, EG2) will receive physical practice of a clinical relevant motor task ('Going down, laying on the floor, and getting up again') over a two week intervention period: EG1 with embedded MI training, EG2 with MI training added after physiotherapy. The CG will receive standard physiotherapy intervention and an additional control intervention not related to MI.

The primary study outcome is the time difference to perform the task from pre to post-intervention. Secondary outcomes include level of help needed, stages of motor task completion, degree of motor impairment, balance ability, fear of falling measure, motivation score, and motor imagery ability score. Four data collection points are proposed: twice during baseline phase, once following the intervention period, and once after a two week follow up. A nested qualitative part should add an important insight into patients' experience and attitudes towards MI. Semi-structured interviews of six to ten patients, who participate in the RCT, will be conducted to investigate patients' previous experience with MI and their expectations towards the MI intervention in the study. Patients will be interviewed prior and after the intervention period.

**Discussion:**

Results will determine whether embedded MI is superior to added MI. Findings of the semi-structured interviews will help to integrate patient's expectations of MI interventions in the design of research studies to improve practical applicability using MI as an adjunct therapy technique.

**Trial registration:**

ClinicalTrials.gov NCT00858910

## Background

Regardless of the decreasing trend in long-term statistics stroke is still one of the three leading causes of death in the US [1] and in Europe. The incidence for individuals older than 55 years range from 4.2 to 11.7 per 1000 [2]. Stroke has a sudden onset resulting from cerebral haemorrhage or ischemia in the brain. In the US, 600'000 first-ever strokes occurred in 2005 with an estimated cost to the community of $ 65 billion in both direct and indirect costs in 2008 [1,3]. In Europe the total costs of stroke are aggregate to 38 € billions in 2006: 49% arise from direct costs, 23% from productivity loss, and 29% from the formal care [4]. Only 25% of the affected individuals recover with minor problems, 45% sustain moderate to severe impairments that necessitate special attention and lifelong care [5].

Several rehabilitation methods address patient recovery, are based on motor learning or neuro-developmental approaches [6-11]. Physiotherapy focuses on regaining motor function, postural alignment and independence in activities of daily living (ADL). Regaining functional ability with a focus on mobility is one of the most frequently targeted short-term rehabilitation goals in patients [12]. New rehabilitation approaches have been reported, e.g. robot-aided [13], virtual reality rehabilitation [14], and MI [15,15]. MI does not require expensive technology, equipment, instrumented locations, and it does not physically exhaust the individual [16]. After initial learning, the MI technique can be practiced by the patient independent from the therapist, location, and time of the day.

The literature on MI techniques has increased tremendously since the late 1990s and there have been several literature reviews related to the application of stroke [17-19]. All found a beneficial value for the recovery of stroke patients when MI was added after physiotherapy or occupational therapy sessions. The analysed randomised or clinical controlled trials used different MI methodologies and compared MI versus an equivalent amount of attention time, where patients listened to information about stroke and relaxation exercises.

MI has its origin in the sports psychology and behavioural psychology in the end of the 19th century [20]. It involves rehearsing a known motor act without any visible muscle contraction or motor output [21]. Several theories are proposed to explain the neuro-physiological mechanisms of MI. The 'psycho neuromuscular theory' was proposed by Jacobson in the early 1930s based on the detection of myoelectrical changes related to the imagined movement [22]. Another theory is based on co-location of brain activation during imagined and real movements in healthy individuals [23-25] as well as in stroke patients [26,27]. Functional magnetic resonance imaging (fMRI) showed activation in frontal, parietal cortical, and sub-cortical areas that are involved in action planning, execution and modulation [25,28]. Recently, the first fMRI study was published that investigated brain activation during imagination of whole body movements [29], supporting the findings from many MI intervention studies in sport psychology.

To date intervention studies in stroke rehabilitation have focused on simple movements, such as reaching and grasping [15,30], whereas investigations in sports psychology used more complex motor skills [31]. Complex motor activities are relevant in rehabilitation as well, since patients seek to regain independence in ADL. Previous stroke research has compared MI to a different mental practice method, e.g. relaxation, or listening to information about stroke. Some researchers have added MI to conventional physiotherapy or occupational sessions [17]. More recently, study proposals were published that will investigate the effect of embedded MI in more complex tasks of daily living [32,33]. Holmes and Collins developed the PETTLEP model to embed MI in training for sports psychology in 2001 [34] based on a combination of seven real-life conditions: physical, environment, task, timing, learning, emotion, and perspective. The PETTLEP model has improved outcome when applied in a MI intervention [35,36]. MI training may be embedded into physiotherapy sessions offering the prospect of more effective and systematic MI training compared to added MI approaches. However, to the authors' knowledge, the PETTLEP model has not been adapted and tested in a stroke population.

Aside the quantitative assessment of MI it is essential to determine patients' experiences with this training method. In sports research the athlete's usage of imagery was assessed with semi-structured interviews, e.g. during rehabilitation of an injury [37] as well as during and after training periods [38]. Driediger et al. [37] reported positive effects of imagery regarding cognitive, motivational, and healing intentions in those athletes. Imagery was used frequently during the day, during rest, e.g. before sleeping, and various other activities such as car driving. Positive and exact images were used to set goals for the rehabilitation process, to handle pain intensity, and to keep a positive stance. These studies found that interviewed athletes were experienced and knowledgeable about imagery and made frequent use of different imagery types, e.g. kinaesthetic, visual, and auditory imagery. Research is needed to transfer these qualitative findings from sports to a stroke population.

### Study aims and research questions

**Figure 1:**
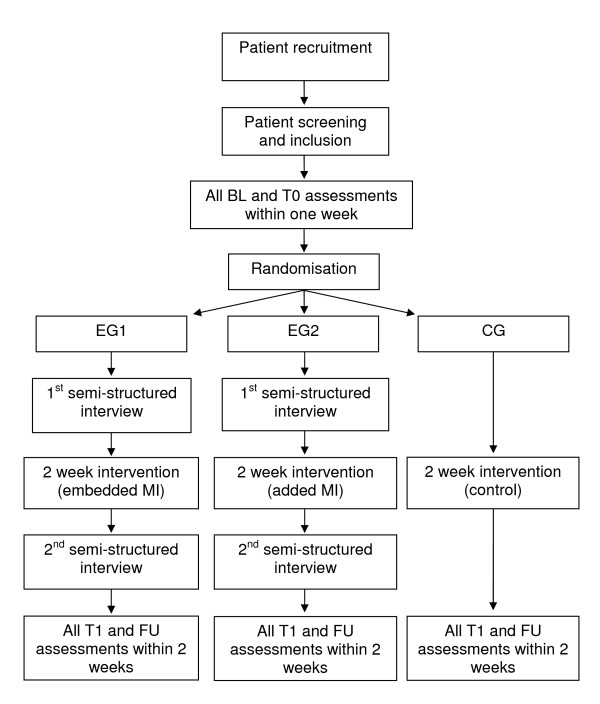
**Study overview**. MI = motor imagery, BL, T0 = 1^st ^and 2^nd ^baseline measurement event, EG1, EG2 = experimental group 1 and 2, CG = control group, T1 = measurement event after the 2 week intervention period, FU = measurement event after a 2 week follow-up period.

This study consists of a quantitative part and a qualitative part. The quantitative part is formed by a therapy intervention with a randomised controlled trial (RCT) study design. The RCT includes three study groups to evaluate the effect MI training embedded into physiotherapy versus MI training added to physiotherapy. In the qualitative part semi-structured interviews of six to ten patients who participate in the RCT will be interviewed to gain knowledge about the patient's experiences and expectations regarding MI. These study parts are compatible to each other and the results are expected to interact. Consequently, the overall study design has been described as a 'mixed methods approach'. Figure 1 illustrates the planned project.

The following sections describe the research protocol of both parts in detail.

#### Aim of the quantitative study part

The aim of the study part is to compare the effect of embedded MI training in physiotherapy (EG1, PETTLEP approach) to added MI (EG2) after regular physiotherapy sessions, regarding improvements of time needed to perform a motor task ('Going down, laying on the floor, and getting up again'). A third patient group with no MI will serve as a control group (CG) to provide an overall baseline for this intervention.

(EG1= experimental group 1, EG2 = experimental group 2)

#### Research question of the quantitative study part

Do patients in the embedded MI training group (EG1) require less time to perform the motor task compared to patients in the added MI training group (EG2)?

#### Aim of the qualitative study part

This qualitative part of the study should add an important insight into patients' experience and attitude to imagery, especially MI. With the help of semi-structured interviews the following aspects will be investigated:

- Before MI intervention:

∘ Patient's previous experience with MI.

∘ Patient's usage of MI regarding the 'W-questions' in imagery research (When, Where, What, Why) [39].

∘ Patient's expectations towards the MI intervention in the RCT.

- After MI intervention:

∘ Patient's attitudes towards the MI after the intervention.

∘ Patient's tentative changes in MI usage.

#### Research question of the qualitative study part

What are the thoughts, feelings, and experience of people with stroke who are participating in MI training? Does it change after a two week MI intervention?

## Methods

### Methodology of the quantitative study part

#### Quantitative study design and setting

In this single-centred randomised controlled trial with a pre-post intervention design, embedded and added MI techniques will be compared to improve a learned motor task ('Going down, laying on the floor, and getting up again'; for a detailed description please see Section 'Motor task and fear of falling on page 13). The study design includes two experimental groups and one control group. Both experimental groups (EG1, EG2) will receive physiotherapy for the motor task: EG1 with an embedded MI training into physiotherapy sessions (PETTLEP approach), EG2 with an added MI training after physiotherapy. The third group is the control group (CG) with physiotherapy and an added control intervention not related to MI. All groups receive the same intervention time of 45 minutes. The investigation will have an intervention period of two weeks and will be carried out in the rehabilitation centre Reha Rheinfelden in Switzerland.

The study has been approved by the responsible cantonal review board of the health department Aarau (Switzerland) and the ethics committee of the School of Health and Social Care at Oxford Brookes University (United Kingdom).

#### Primary and secondary outcome measures

Table 1 provides an overview on all study outcome measures. All outcome measures will be assessed at four times: twice during the baseline phase (BL, T0), after the intervention (T1), and after the two week follow-up phase (FU). Changes over time will be calculated by the differences between BL vs. T0, T0 vs. T1 and T1 vs. FU.

**Table 1:** Overview study endpoints and outcome measures

	**Primary outcome**	**Primary outcome measure:**
	Time difference between T0 and T1 needed to perform the motor task ('Going down, laying on the floor, and getting up again')	Time will be given in seconds and will be obtained by the recorded video of the task performance.

	**Secondary outcome**	**Secondary outcome measures**

**1**	Time difference between BL vs. T0 and between T1 vs. FU	Time will be given in seconds and will be obtained by the recorded video of the task performance.

**2**	Help needed to perform the motor task	Assistance levels of the activity scale of the Chedoke McMaster Stroke Assessment (CMSA)

**3**	Stage of the motor task	Stage of the motor task by Bergland and Lake will be obtained by the recorded video of the task performance.

**4**	Imagination inflation	Patients will be asked before each motor task execution during the assessments to predict time they expect to need to perform the motor task.

**5**	Motor impairment and independence	German version of the extended Barthel index (EBI)

**6**	Balance	German version of the Berg Balance Scale (BBS)

**7**	Motor imagery ability	German version of the kinesthetic and visual motor imagery questionnaire (KVIQ-G)

**8**	Understanding of the motor imagery instructions	German version of the Imaprax software version 1.1

**9**	Fear of falling (FOF)	German version of the Activities-Specific Balance Confidence Scale (ABC-scale)

**10**	Intrinsic motivation	Motor imagery diary

**11**	Mood	Direct question that can be answered on an 11-point VAS: 'How sad do you feel today?'

All outcome measures will be assessed at four times to measure changes over time: twice during the baseline phase, after the intervention, and after the two week follow-up phase.

##### Primary outcome measure

The primary study outcome has been defined as changes in time needed to perform the motor task due to the study intervention. This primary outcome will be assessed by the difference between measurement points T0 and T1. For performance and validity reasons all assessment sessions will be videotaped. Time needed to perform the task in seconds will be derived from the video recordings.

##### Secondary outcome measures

Secondary outcome measures have been categorised into four different profiles that utilise distinctive assessment types.

##### Motor task related profile

1. Time difference to perform the motor task between BL and T0 as well as between T1 and FU.

2. Help needed to perform the motor task will be evaluated using classification levels of the activity scale of the Chedoke McMaster Stroke Assessment (CMSA) [40,41]. The CMSA is a validated and very reliable performance-based assessment [42-44]. The activity scale belongs to the gross function index and is scored with one (patient needs total assistance or the task is not tested for safety reasons) to seven (patient can perform the task completely independent without any assistance or devices, in a reasonable time) on an ordinal scale [45].

3. Stage of the motor task will be evaluated using the classification of Bergland and Laake [46] (please see section 'Stages of the motor task').

4. 'Imagination inflation': Undergraduate college students overestimated their motor performance after MI training. This effect is called 'Imagination inflation' and was detected by Landau et al [47]. To monitor the inflation in this investigation all patients will be asked before each motor task execution during the assessments to predict time they will need to perform the task before each motor task execution.

##### Motor impairment and balance profile

The motor impairment and balance profile will be evaluated with the German versions of the extended Barthel index (EBI) and the Berg Balance Scale (BBS). Both assessments will take about 15 to 20 minutes each to complete.

5. The EBI is a 16-item performance-based measurement. Activities of daily living (ADL), e.g. personal hygiene, dressing, feeding, and cognitive aspects, e.g. communication and problem solving, will be scored on a five-point Likert scale (0 = cannot perform the task, 4 = independent) [42,48]. The total score will be used to evaluate trends. Validity and reliability were examined with patients after stroke and multiple sclerosis [49-52]. A validated German version is available [50].

6. The BBS consists of 14 items to assess two dimensions of balance impairments in (older) individuals and patients undergoing rehabilitation. The ability to maintain an upright posture in different positions and the ability to adjust posture when reducing support surface will be scored on a 5-point ordinal scale (0 to 4). High scores are interpreted as a sufficient ability to perform a task or to perform it within a given time frame [42,53]. The total score will be used to evaluate trends. Validity, reliability and sensitivity to change were evaluated [53-56]. A validated German version is available [57].

##### Motor imagery profile

The motor imagery profile will be assessed with the Kinesthetic and Visual Imagery Questionnaire (KVIQ) and the Imaprax software, version 1.1. The completion of both assessments will take 30 to 40 minutes.

7. The KVIQ includes a visual and a kinaesthetic imagery scale and was developed based on the revised movement imagery questionnaire (MIQ) [58]. The new measure was specifically developed to assess motor imagery ability for individuals with motor impairments in 2007 by Malouin et al. [59]. The questionnaire is available in a short (10 items) and a long version (20 items). The latter will be used in this investigation. All items will be evaluated in a standardised sequence while sitting for visual and kinaesthetic scales. The assessment requires performing a sequence of simple movements once, imagine the movement once and score the vividness of the 'inner picture' on a 5 point Likert scale (1 - 'image as clear as actually seeing it' to 5 'no image') as well as the feeling of the imagined movements (1 - 'as intense as making the movement' to 5 - 'no sensation'). The total score of each subscale will be used to evaluate trends.

8. The Imaprax 1.1 software was developed to evaluate understanding of MI, vividness of movement imagery, and imagery perspective in patients with apraxia following stroke. It is based on software that was used with skydivers [60,61]. Six gestures or activities will be evaluated in a three step standardised procedure: select the correct gesture or activity from three proposed ones, evaluate the vividness of the 'inner picture', and determine the internal or external perspective of your 'inner picture'. The total score of MI vividness will be used to evaluate trends.

KVIQ and Imaprax assessment tools were published in other languages than German. Following a cross-cultural translation and adaptation based on guidelines for self-report questionnaires by Beaton et al. [62] both assessments were forward translated into German. To check for consistency both tools were backward translated into their original publication languages and checked by the original authors. We obtained the permission from those authors to use the translated assessments in our study.

##### Psychological profile

The psychological effect of MI is not yet proven. Nevertheless, authors of published MI studies proposed to include assessments of selected psychological factors, e.g. attention and working memory [63]. The psychological profile includes the evaluation of patient's fear of falling (FOF) using the Activities-Specific Balance Confidence Scale (ABC-scale).

9. The ABC-scale is 16-item questionnaire that evaluates the self-confidence of a person based on Bandura's theory of self-efficacy [42,64]. It can be self-administered or completed during a face to face interview. Patients determine their self-perceived confidence to remain in balance on a visual analogue scale (VAS) ranging from zero to 100 percent (10 cm). The mean value will be used to evaluate trends. The scale is a valid and reliable assessment that is available in German language [65,66].

10. Furthermore, intrinsic motivation will be evaluated from the patient's MI diary. Using details on frequency of independent MI practice reported in the patient's diary motivation to practice and the compliance with the training can be determined [67]. Other investigations showed that patients regained self-control about their recovery process. They felt more skilful, and patients as well as athletes expressed their satisfaction and belief in the MI training, which helped to improve their performance [68,69].

11. Patient's mood will be enquired by a direct question: 'How do you feel today?'. This will be scored on an 11-point VAS ranging from zero (very good) to ten (very bad).

##### Further evaluations and assessments

To further describe the included study population the following descriptive data will be obtained from each participant: age, gender, weight, disease, affected side, time since stroke onset, actual medication, cognitive function, handedness, rehabilitation history (treatments until study start), patient's sports history, and patient's history of falls since the stroke onset (over the last six to twelve months). Data will be obtained by direct questions to patients, from medical records, and from a relative or proxy.

Cognitive function will be assessed with the Mini-Mental State Examination (MMSE). MMSE is a short screening tool for dementia symptoms [70]. MMSE includes items to evaluate spatial and temporal orientation, short-term memory, attention and calculation abilities, language, thinking, and action planning. The total score is 30 points. MMSE will be performed once at a baseline assessment and will take about 15 to 20 minutes.

Patient's handedness will be determined with the Edinburgh Handedness Inventory (EHI) once. The EHI is a valid and reliable short 10-item questionnaire. Patients will be asked what is the preferred hand to carry out activities of daily living (ADL), e. G. Using toothbrush, cut something with a scissor, or use a spoon [71]. The EHI will take about five to ten minutes to complete.

Further important information that may influence the effect of MI are:

- patient's type of learning (visual or kinaesthetic),

- patient's belief in the therapy [72].

Both will be assessed by direct questions during baseline measurement events.

#### Recruitment process and patient selection criteria

Patients will be recruited according to the following procedure:

1. A procedure common in retrospective research will be used. Former inpatients of the rehabilitation centre with a cerebrovascular ischaemia or a hemorrhagic bleeding will receive an information letter including a prepared answer sheet and a paid envelope. When interested in receiving more information patients may return the envelope with the answer sheet or contact the project leader by telephone or email.

2. Upon receiving answer sheets back in the rehabilitation centre, patients will be sent the patient information sheet to inform them about the planned study.

3. Three to ten days after sending the patient information sheet, patients will be contacted by phone to ask, if they have received the patient letter and if they have any questions they would like to have answered. If the patient is interested, a first appointment will be arranged to provide detailed information about the study procedure and check for study eligibility. This appointment will be arranged at rehabilitation centre, the patient's home or institution.

Additionally, patients will be recruited as leaving inpatients or from the outpatient therapy department of the rehabilitation centre by their treating therapist, through an advertisement at the centre's homepage, and through flyers at several locations of the centre. Interested patients can contact the project leader through their therapist, by phone, email, or mail. Patients will be selected based on the criteria in Table 2.

**Table 2:** Patient inclusion and exclusion criteria

**Inclusion criteria**	**Exclusion criteria**
- Patients after a first-ever ischemic or hemorrhagic stroke - Outpatients or inpatients 3 months after stroke - Ability to stand with or without a cane for at least 30 sec on a normal hard floor - Ability to walk for 20 metres with or without a cane or an orthosis - MMSE with at least 20 points - Age older than 18 years - Signed written informed consent	- Joint replacements (knee, hip, shoulder) - Limiting pain in the upper or lower body (evaluated with an eleven point VAS rating scale) - Limiting range of motion (ROM) in the hip, knee, ankle joint or toes - Body weight more than 90 kilograms - Compromised mental capacity to give written informed consent

#### Study procedure and measurement events

The study procedure and all measurement events are illustrated in Figure 1. After signing informed consent, patients will start with the baseline phase that extends over week 1 and week 2. During this time patients will undergo all clinical measurements twice (BL, T0) at an interval of six to seven days. After the baseline phase all patients will be randomised to one of three study groups: EG1, EG2, or CG. Each group will receive three therapy sessions per week for two weeks, hence six sessions in total. Each session lasts 45 minutes. Based on the main study hypothesis, therapy for EG1 will embed MI training during physiotherapy, while EG2 will receive MI training after the physiotherapy part. For a detailed description please see Section 'Study intervention' on page 14. Baseline measurements will be repeated directly after the therapy session weeks to evaluate short-term effects of the MI intervention (T1), as well as after a follow up period of two weeks (FU). All intervention and assessment sessions will take place at the rehabilitation centre's physiotherapy department.

#### Randomisation and allocation concealment

Patients will be randomly allocated to the study groups (EG1, EG2, CG) to ensure internal validity of results. Group allocation to one of the three study groups will be performed after the second baseline assessment (T0) using a randomisation list. The list has been generated with MATLAB 2007b (Mathworks Inc., USA) by a researcher not involved in the study. The generated list has been sent to the clinic's pharmacist, who is not involved in the study and will have no contact with study patients. The pharmacist will prepare sealed envelopes with the patient allocation based on the randomisation list. Patients will be given the sealed envelope by the treating therapist and they will open the envelop themselves. After opening, envelopes will be stored with the patient's personal documents in a locked cabinet. Only the treating therapist and the research assistant will have access to the documents. The independent examiner will not have access to the documents. Patients will be told not to talk to the examiner about the group allocation or therapy content during the post-intervention assessments. Randomisation will be concealed to the independent examiner until the last follow-up assessment of the patient has been performed.

#### Blinding and study group interaction

Blinding of study personnel in research projects is a main quality criterion of a study [73]. Nevertheless, blinding of therapists in an intervention study who perform experimental treatment in patients is not always feasible, especially if experimental intervention shall be integrated in the therapy. Consequently, hiding the patient's allocation in this setting will not be possible. Therefore, neither therapists nor patients will be blinded in this study. Nevertheless, the assessor of post-intervention and follow-up assessments and the independent statistician who will control statistical analyses will be blinded to patient's group allocation until all assessments have been performed. Interaction of patients with other study participants will be minimised by the following preventive measures:

- The patients will be asked to not speak about their study and therapy content as well as imagery sessions outside their families, and neither to talk to the assessor about the study and session content.

- All examiners will be instructed not to ask patients about their study content.

- All patients are outpatients, coming to the rehabilitation centre for the study intervention only. They do not stay together in one clinic ward.

- Group sessions will be avoided.

#### Motor task and fear of falling

The motor task 'Going down, laying on the floor, and getting up again' is clinically relevant, in particular for older people who have problems with ambulation and balance. This task is an important skill to live independently and maintain activities of daily living (ADL) [46,66]. A fall is defined as an involuntary position alteration that results in laying on the floor or ground. It can cause injuries such as fractures, soft tissue injury or joint dislocations [46,74]. In particular, lacking the ability to get up from the floor is related to fear of falling (FOF) in the elderly [46]. FOF is a psychological construct that helps to estimate an individual's ability to perform ADL without falling [66]. Friedman and colleagues found that FOF can emerge from an experienced fall and it can exist within non-fallers [75]. Patients after stroke have a 2.3 times higher risk of falling than the age-matched normal population [76]. This risk can increase up to 3.4 times if more than one fall already occurred. It is known that a majority of falls happen during day time, indoors as well as outdoors, while walking, and while changing the body position, e.g. from sitting to standing [76]. These findings confirm the relevance of the selected motor task in this intervention study. Hence, it is expected that MI training and physiotherapy sessions will reduce FOF and improve patient's self-confidence in walking.

##### Stages of the motor task

The stages of the motor task are a modified version from the task stages of Bergland and Laake [46]. They proposed a movement sequence with 13 stages that is helpful for training with elderly individuals having FOF. The patient stands facing a mat on the floor. After the instruction 'Get down to the floor mat' the patient moves down to the mat in a standardised procedure to lie straight on the mat with legs extended. After the command 'Stand up' the patient stands up, moving from the laying position on the floor to an upright position by repeating all stages in reverse order. Two stages were modified: the starting position (stride standing) will be included as the first stage because this stride standing is already challenging for patients after stroke. The original stage 5 (to prone kneeling and up) will be left out because only a small portion of patients after stroke are able to bear the trunk weight at the affected hand, arm, and shoulder while maintaining the arm in an extended position.

Motor task stages considered in this study

- Stage 0 - Standing

- Stage 1 - Stride standing

- Stage 2 - To half-kneeling on to a large foam wedge and up

- Stage 3 - To half-kneeling on to a small wedge and up

- Stage 4 - To half-kneeling on a mat and up

- Stage 5 - To high-kneeling on a mat and up

- Stage 6 - To half-sitting on two pillows and up

- Stage 7 - To half-sitting on one pillow and up

- Stage 8 - To half-sitting on a mat and up

- Stage 9 - Side laying on a large wedge and up

- Stage 10 - Side laying on a small wedge and up

- Stage 11 - Side laying on a mat and up

- Stage 12 - Supine laying on a mat and up

The motor task from Bergland and Lake [46] for elderly people contains thirteen stages and the highest level represents the highest level of motor task completion. This classification will be maintained for the modified motor task in this study with stroke patients.

#### Study intervention

##### Therapy session and physiotherapy

All groups receive physiotherapy treatment based on a mixed neuro-physiological and motor learning approach three times a week for two weeks [77]. Patients will be treated by an experienced physiotherapist with a working experience of at least two years in neurological rehabilitation. Each session will include activities while sitting and walking depending on the motor impairment level of the patient. The main content of the sessions will focus on exercises and activities to improve postural control in different starting positions, preferable positions (or surfaces) with small support to bear body weight (e.g. sitting, standing). The motor task 'Going down, laying on the floor, and getting up again' will be practised once during physiotherapy in all study groups. To maintain an equivalent practice level, patients will be asked not to practice the motor task at home during the intervention period.

In the therapy sessions it is not allowed to:

- practice the motor task more than once,

- practice the motor task in a different order,

- practise parts of the motor task on a treatment bench.

##### Embedded motor imagery training (EG1)

The MI intervention will be embedded into physiotherapy of the six therapy sessions which last for 45 minutes each. For EG1 the complete motor task will be divided into its thirteen stages. Each stage will be mentally rehearsed before and after it is once physically practised.

The embedded MI procedure is based on the PETTLEP approach [34] that can be summarised as follows:

- **Physical/Emotion**: Imagination of the motor act where it should be performed, without any prior relaxation exercises, in an active and alert state.

- **Timing**: Duration of the motor task should not exceed the real performance duration.

- **Environment**: Using (personalised) multisensory environmental cues.

- **Task/Learning/Perspective**: Depending on the patient's learning type and its familiarisation with the task, external or internal perspective is chosen.

Patients will be encouraged to rehearse the motor task mentally as often as possible between therapy sessions. They will be asked to keep a diary of their individual mental rehearsals to measure rehearsal frequency.

##### Added motor imagery training (EG2)

After 30 minutes of physiotherapy in each therapy session the participants of EG2 will receive an added MI training. This training will be based on the studies of Page et al. [30,78,79]. Patients will listen to a tape that consists of three parts: part one is a brief relaxation period up to three minutes, afterwards in part two, patients listen to the description of each motor task step that should be imagined, and finally, in part three, patients will have a short period to refocus on the room and the situation (two minutes). Patients will listen to the tape in a separate quiet room in a supine laying position on a treatment bench. As in EG1, participants of EG2 will be encouraged to rehearse the motor task mentally as often as possible between therapy sessions. They will be asked to keep a diary of their individual mental rehearsals.

##### Control group (CG)

Besides receiving physiotherapy during a 30 minutes session, participants in the CG will listen to a tape lasting for 15 minutes. The rationale for the tape is to provide in CG participants with the same therapeutic attention as applied in EG1 and EG2. It is important that the participants do not imagine movements. Therefore, the tape will start with a short relaxation period up to three minutes. Afterwards patients will listen to information about stroke: its cause, its consequences for different body functions and its recovery phase, therapy options, prevention of potential complications, self-help groups and their offers. This control protocol has been used in other MI studies without negative effect reported by authors [30,80]. All tapes will have an encouraging character and patients will be asked how they liked the information on the tape. Patients will listen to the tape in a separate quiet room in a supine laying position on a treatment bench.

The CG is a very vital aspect of this pilot study to show a treatment effect of both experimental groups versus a CG [81-83]. Since the PETTLEP approach has been investigated in athletes, its efficacy in a stroke population is not known. Therefore, a CG helps to determine the benefit of embedded MI following the PETTLEP approach in a stroke population. Furthermore, if no difference between both experimental groups can be detected a comparison with the CG will show the overall effect of the MI intervention. Without this option no conclusion could be drawn from the experimental intervention and the scientific value of the investigation would be diminished.

Embedded MI (as in EG1) is the novel MI approach investigated in this study. Added MI (as in EG2) is the current MI therapy standard against EG1 will be benchmarked. The investigated methodologies result in different therapist contact times for all study groups. To nevertheless compare all groups in this study, a methodology was implemented to minimise the effect of different therapist contact times. Our approach is to embed the tape listening (EG2, CG) into times of patient-therapist contact and cue the patient involvement. In particular, the following measures have been taken:

- The tape used in EG2 and the CG has been recorded with the research therapist guiding the patient. Hence patients hear the same voice from the tape as they are used in the physiotherapy training.

- While listening to the instructions from the tape, patients in EG2 will be asked to imagine the complete motor task and to report the number of motor task imaginations to the research therapist afterwards. That request has been included to remain concentrated and attentive during the added MI session.

- Before and after listening to the tape (EG2, CG), the research therapist will be present in the room, to help patients in sitting up and put on/off cloths. The therapist will ask patients how they liked the tape content.

#### Statistical analyses

All necessary statistical analyses will be performed with the Statistical Package for the Social Sciences (SPSS) version 16. The significance level will be set at p ≤ 0.05. For all outcome measures the final analysis method will be determined after data collection tests of normal distribution and homogeneity of variances. Before data analyses collected patient data will be anonymised.

##### Sample size calculation

This proposed pilot study is a phase II intervention study and the first of its kind in the field of neurological rehabilitation comparing two MI intervention techniques. Both intervention techniques were tested separately in randomised controlled trials with stroke patients or athletes. As the hypothesis of this investigation has not been addressed before, an a priori sample size cannot be estimated.

##### Group comparison

Group equality will be determined at baseline regarding all descriptive variables: age, gender, disease, affected brain hemisphere, time since stroke onset, cognitive function, handedness, rehabilitation history, patient's sports history, and patient's history of falls since stroke onset (over the last six to twelve months). Parametric and nonparametric tests will be used depending on statistical data level and data distribution. Furthermore, inter-group comparisons between BL and T0, T0 and T1, as well as T1 and FU will be calculated to analyse changes among all three groups over time. Additionally, intra-group comparisons for each measurement event (pre- and post-intervention; T0 vs. T1 vs. FU) will be calculated for all three groups; means, standard deviations and confidence intervals, will be given where feasible. An intention to treat analysis will be performed for drop outs. Missing values will be replaced by the average trend of all participants of the respective group.

#### Presentation of results

The patient recruitment process, the total number of included and excluded patients, as well as the dropout rate will be summarised in a CONSORT flow diagram [84]. All data describing the study population (general profile) will be presented in an overview table. The changes between assessment events and differences between study groups will be displayed in graphs and tables. Significant changes will be marked. Standard deviations and confidence intervals will be used to describe dispersion characteristics.

### Methodology of the qualitative study part

Our exploratory approach applied in the qualitative part evaluates patient's prior experience and usage of MI as well experience they have gained during the study intervention. Current literature did not consider stroke patient's experience obtained with MI. In consequence it is not clear how patients can use MI. As the MI techniques are not yes standardized, no assessment on user experience of MI exists. We have chosen semi-structured interviews to obtain an important insight into patient's experience and attitudes to MI. Additionally, new hypotheses for further investigations can be derived. Embedding MI into physiotherapy is a new therapy approach. The patient's awareness and positive or negative experience during the embedded intervention could be explored in detail during the semi-structured interviews. The qualitative approach allows to discover potentially vital aspects promoting or inhibiting the MI execution. Insight gained from the interviews can help to adapt the MI approach to patient's needs. This may have a critical impact on the further development of MI techniques, in particular, on follow up studies after this pilot trial.

Semi-structured interviews will be conducted twice for this nested qualitative research study. Patients will be interviewed after randomisation to one of the two experimental groups of the RCT and after the last MI intervention session.

#### Sampling and comparison

The inclusion and exclusion criteria of the RCT (please see Table2) describe main characteristics of the patients. For the qualitative part, six to ten patients will be recruited from the whole study sample. Regarding the diversity and heterogeneity of the sample, the following additional criteria will be considered:

- Ability to speak and express thoughts and feelings.

- Inclusion of both genders.

- Inclusion of different ages (younger and older patients).

- Inclusion of patients at different stages of the recovery process (sub-acute or chronic stage).

- Inclusion of patients with different levels of motor impairment.

- Inclusion of different kinds of professions.

- Inclusion of different amount/level of sports before stroke.

- Willingness to participate in the interviews and signed informed consent.

At least three statements of the interviewees will be compared among each other. If possible, a group comparison analysis will be included based on the patient's time since stroke onset.

#### Data collection

After randomisation to one of the experimental groups, patients will be given an additional information sheet and a separate consent form. After receiving informed consent from the participants, data will be collected during semi-structured interviews. The interviews can take place either in the rehabilitation centre or at the patient's home. It is important to create a relaxed and quiet atmosphere. Interviewee and interviewer will sit next to each other on a sofa or chairs close to a table. Interviews will be recorded with a digital voice recorder. The interviewer will take notes during the interview to picture the scenario. These notes will provide the basis for field notes that will be written by the interviewer directly after each interview. Field notes will include information on interview situation; the patient's acting during the interview, her/his facial expressions, gestures, mood, feelings, and course of the interview. Further information about unexpected events or statements, feelings, and impression of the interviewer will be included as well. Depending on fatigue and concentration ability of the patient, interviews will last between 30 minutes up to one hour.

##### Interview guide and questions

The interview can be seen as an active inter-action of two people. Regardless of the prepared interview questions, the interviewer will react on the patient's statements. The interviewer has to formulate spontaneous questions to follow-up answers into more detail.

The interview is divided into three parts: introduction, main and final parts. During the introduction patients will be familiarised with the interview procedure. After starting the recording participants will be asked if she/he has any open questions regarding the information sheet or the informed consent. Additionally, the interviewer will point out that the recording or the whole interview can be stopped at any time if the participant wishes to do so. The introductory part includes broad start questions regarding the stroke event, recovery process, and rehabilitation phase up to this point. Furthermore, interviewees will be asked about previously occurred falls, their fear of falling, and their coping strategies. The participants will be encouraged to talk about their impressions and feelings.

The main part of the interview will focus on the patient's experience with MI. Interviewees will be asked

- what individual experiences they have in learning and using MI,

- what MI means to them,

- what exactly do they imagine,

- when do they use MI,

- why they use MI and

- where do they perform MI.

Additionally, to check for patient's understanding, all participants have to perform and imagine the task 'Standing up from the chair and sitting down'. After performing the task, interviewees will be asked: what they have imagined, how did it feel to imagine, how did they like it, what they think about MI therapy, what they expect from the study intervention, and how MI could help them.

Moreover, time needed for the physical and imagined activity performance will be compared to check for congruence of activity duration. To assess activity duration participants will time physical and imagined activity themselves with a stopwatch. In the last part of the interview the interviewer will briefly summarise patient's statements. Participants will be asked if they would like to make additional comments to anything they have said. Furthermore, interviewees can talk about their impressions from the interview (situation, questions). They can make suggestions, express critique or encouragement. All important statements of patients after stopping recordings will be noted by the interviewer during or after the interview and will be included in field notes.

Semi-structured interviews after the two week intervention period will mainly focus on the experience of patients during this time and, if their usage of MI and their attitude towards MI have changed.

All interview questions were developed based on the findings from Munroe et al. [39] and the interview guide from MacIntyre et al. [38].

#### Transcribing verbal data

All recorded data will be verbatim transcribed by a research assistant with good typewriting skills and with the help of the f4 software for digital transcriptions [85]. As there exists no defined standard to transcribe verbal data, the research assistant will include pauses and repetitions during the interview and will follow the transcription suggestions of Kvale [86] and Bortz [87]. The text will be checked for congruity with the audio data by the research assistant who did the transcription and double-checked by the interviewer. The transcription documents will be complemented by detailed field notes of the interviewer and notes of the analysis process. In combination, these documents will provide a picture of the interview situation and will provide the basis for data analysis.

#### Data analysis

Kvale [86] suggests to start with the analysis already during conduction of interviews. Interviewers should ask 'second questions' during interviews to gain more detailed knowledge about the meanings of patient's statements, e.g. 'Can you tell me more about that?' or 'Could you give me some examples for that?'. Another technique is to condense and summarise statements of the interviewee and check back for correct interpretation directly during the interview. Both techniques will be applied in this qualitative part. Before starting the analysis all documents will be quality checked. To gain an overview on the interviewees and their statements, short case descriptions will be written. These short notes will include descriptive characteristics of the interviewee and main interview topics with suitable quotations. The main analyses of the interview will be done with the Nvivo software [88]. Data collection and data analysis will be based on a phenomenological approach to build a structure from which themes will emerge (thematic analysis). The interview material will be categorized and coded using both, software and personal immersion in the data following Gibbs' recommended approach [89].

#### Quality issues

Transparency of the interview and analysis process is one major opportunity to increase quality in qualitative research and allows a stepwise replication of the research process. Transparency is achieved in this study by documenting 'second questions' during the interviews and maintaining a process for categorisation and coding of transcribed interviews.

An essential approach to increase research quality is to plan and conduct investigations based on trustworthiness, which includes credibility and transferability. Credibility will be addressed in this study by member validation and peer validation [86]. Member validation aims to decrease misinterpretation of interviewees and provides them with the opportunity to comment on the data interpretation. All interviewed participants will be given the opportunity to read the themed analysis to confirm the resource of the themes with their own experiences that have emerged to confirm the validity of the data. Peer examination helps the interviewer to discuss data interpretation with other colleagues and get feedback on the analysis. A third approach to improve quality aspects is the mixed methods design of the research proposal. Quantitative data, e.g. of the KVIQ, can support the qualitative analysis. Triangulation of both methods will provide complementary information and an in-depth understanding of the patient's attitude towards MI and benefits from the intervention study.

#### Presentation of analysis

The analysis will include the common structure of a journal article: introduction/background, methods, results, discussion, and conclusion, adapted to the journal's instructions for authors. Extracts from the transcribed material will be included in the article and will be embedded into quotation marks. The categorisation scheme and the category definitions will be included in the appendix of the publication.

## Discussion

This mixed methods protocol describes an intervention pilot study proposal aiming to compare two different MI techniques in patients after stroke: the sports psychology approach of an embedded MI training into physiotherapy (based on the PETTLEP model) and added MI training after physiotherapy. The third group serves as a control to evaluate the effect of MI vs. A control intervention. A qualitative was integrated into the protocol to gain in-depth knowledge of participant's attitude towards MI and modifications in MI usage during the intervention study.

Findings from fMRI studies provide the neuro-physiological basis of current MI training interventions. Brain areas activated during MI and real movements show a strong congruity for single arm movements as well as complex whole body movements in stroke patients [26,29]. Similar findings were made for other neurological disorders e.g. Mb. Parkinson and amyotrophic lateral sclerosis [90,91]. Intervention studies confirmed a beneficial effect of MI in patients after stroke. Moreover, these results were confirmed in further patient groups, including traumatic brain injury, multiple sclerosis, and Mb. Parkinson [92-94].

The PETTLEP approach was developed for performance improvement of athletes and describes seven important conditions that should be considered during MI training. To this end, the PETTLEP model provides a systematic and embedded approach to MI training. In contrast to PETTLEP, the approach established in stroke rehabilitation adds MI to therapy sessions by including a relaxation phase between physiotherapy and MI training. This added MI training was neither performed in the actual physical training environment nor in the task-specific body position, as is requested for PETTLEP [34]. Based on a preliminary analysis and the current study design we expect that the PETTLEP model can be transferred to stroke rehabilitation.

The perspective that is taken by participants during MI interventions is a controversially discussed issue [34]. Which perspective is the right one? External, hence the participant watching herself/himself or another person performing a task in front of her/his inner eye, or internal, where the participant watching her/his arm or leg from own eye perspective? Do all patients choose the same perspective at the beginning? Does the perspective selection depend on the level of experience with a particular motor task? By determining the learning type and the preferred self-selected perspective the authors hope to contribute towards resolving this debate.

The complex motor task 'Going down, laying on the floor, and getting up again' was chosen for two reasons: firstly, it is clinical important to learn how to get up from the floor, and secondly it is psychologically relevant for all individuals with the risk of falling (fear of falling), in particular for patients after stroke. The study will furthermore provide essential results to determine the benefit of MI as similarly complex motor tasks have not been investigated in stroke rehabilitation before.

Three outcome measures (time and help needed to perform the task as well as level of task completion) will be supplemented by psychological assessment of FOF and motor imagery understanding and ability assessment. Taken together, all assessments will draw a comprehensive picture of a patient's capabilities and subsequent changes during the intervention and follow up period. The motor task assessments will be video recorded. From analysis of assessment videos, it is expected to obtain a more detailed description of the motor task stages, quality of movements, and impact of MI training on the motor task. Furthermore, time ratio of imagination and performance of the motor task will be calculated from patient prediction and actual scores to evaluate the 'Imagination inflation' [47]. These results will provide information about the patient's therapy coherence and acceptance of MI.

Duration of the intervention period was defined after considering MI studies in stroke rehabilitation as well as in sports psychology. In sports psychology, very short MI treatment durations were chosen in comparison to rehabilitation [95]. The duration in this study design is shorter than that of added MI interventions, since the approach using MI embedded in standard physiotherapy requires less time. However, it is expected that the embedding approach will compensate for the reduced duration.

A comparison of embedded and added approaches to MI training in a stroke population sample will contribute to a broader understanding and more focused design of MI interventions in stroke patients. Results will help to answer the question on which MI training approach is more beneficial for patients after stroke and whether a sport psychology model can be transferred directly to rehabilitation practice. Findings from the semi-structured interviews will help to integrate patient's expectations on MI interventions in the design of research studies to improve practical applicability.

## Abbreviations

ABC-Scale: Activities-Specific Balance Confidence Scale; ADL: Activities of daily living; BL - 1st Baseline measurement event; BBS: Berg Balance Scale; CG: Control group; CMSA: Chedoke-McMaster Stroke Assessment; EBI: Extended Barthel Index; EG1: Experimental group 1; EG2: Experimental group 2; EHI: Edinburgh Handedness Inventory; fMRI: Functional magnetic resonance imaging; FOF: Fear of falling; FU: Follow up; KVIQ-G: Kinesthetic and Visual Imagery Questionnaire - German version; MI: Motor imagery; MIQ: Revised Movement Imagery Questionnaire; RCT: Randomised controlled trial; ROM: Range of motion; SPSS: Statistical package for social sciences; T0: 2^nd ^baseline measurement event; T1: Post-intervention measurement event; VAS: Visual analogue scale.

## Competing interests

The authors declare that they have no competing interests.

## Authors' contributions

CS, JB, BA and UK participated in the design of the study and helped to draft the manuscript. CS and TE are the leading researchers at the trial site. All authors helped to draft the manuscript and approved the final version of the manuscript.

## References

[B1] Kung H-C, Hoyert DL, Xu J, Murphy SL (2008). Deaths: Final Data for 2005. National Vital Statistics Report.

[B2] Feigin VL, Lawes CM, Bennett DA, Anderson CS (2003). Stroke epidemiology: a review of population-based studies of incidence, prevalence, and case-fatality in the late 20th century. Lancet neurology.

[B3] Sosulski MR, Lawrence C (2008). Mixing Methods for Full-Strength Results: Two Welfare Studies. Journal of Mixed Methods Research.

[B4] Allender S, Scarborough P, Peto V, Rayner M, Leal J, Luengo-Fernandez R, Gray A (2008). European cardiovascular disease statistics.

[B5] Rehabiliation Therapy. http://www.stroke.org/site/PageServer?pagename=REHABT.

[B6] Tyson SF, Selley AB (2007). The effect of perceived adherence to the Bobath concept on physiotherapists' choice of intervention used to treat postural control after stroke. Disabil Rehabil.

[B7] Taub E, Uswatte G, King DK, Morris D, Crago JE, Chatterjee A (2006). A placebo-controlled trial of constraint-induced movement therapy for upper extremity after stroke. Stroke.

[B8] Khadilkar A, Phillips K, Jean N, Lamothe C, Milne S, Sarnecka J (2006). Ottawa panel evidence-based clinical practice guidelines for post-stroke rehabilitation. Top Stroke Rehabil.

[B9] Ada L, Dorsch S, Canning CG (2006). Strengthening interventions increase strength and improve activity after stroke: a systematic review. Aust J Physiother.

[B10] Woldag H (2005). Modern therapeutic approaches in the rehabilitation of walking ability after stroke. Stroke.

[B11] van Vliet PM, Lincoln NB, Foxall A (2005). Comparison of Bobath based and movement science based treatment for stroke: a randomised controlled trial. J Neurol Neurosurg Psychiatry.

[B12] Geschwindner HM, Rettke H, Heuvel WJ van den, Halfens RJ, Dassen T (2007). Rehabilitation in acute stroke patients in German-speaking Switzerland. Swiss Med Wkly.

[B13] Mayr A, Kofler M, Quirbach E, Matzak H, Frohlich K, Saltuari L (2007). Prospective, blinded, randomized crossover study of gait rehabilitation in stroke patients using the Lokomat gait orthosis. Neurorehabil Neural Repair.

[B14] Eng K, Siekierka E, Pyk P, Chevrier E, Hauser Y, Cameirao M, Holper L, Hagni K, Zimmerli L, Duff A (2007). Interactive visuo-motor therapy system for stroke rehabilitation. Med Biol Eng Comput.

[B15] Liu KP, Chan CC, Lee TM, Hui-Chan CW (2004). Mental imagery for promoting relearning for people after stroke: a randomized controlled trial. Arch Phys Med Rehabil.

[B16] Jackson PL, Doyon J, Richards CL, Malouin F (2004). The efficacy of combined physical and mental practice in the learning of a foot-sequence task after stroke: a case report. Neurorehabil Neural Repair.

[B17] Zimmermann-Schlatter A, Schuster C, Puhan M, Siekierka E, Steurer J (2008). Efficacy of Motor Imagery in post-stroke rehabilitation: a systematic review. J NeuroEng Rehabil.

[B18] Braun SM, Beurskens AJ, Borm PJ, Schack T, Wade DT (2006). The effects of mental practice in stroke rehabilitation: a systematic review. Arch Phys Med Rehabil.

[B19] Sharma N, Pomeroy VM, Baron JC (2006). Motor imagery - A backdoor to the motor system after stroke?. Stroke.

[B20] Jastrow JA (1892). Study of involuntary movements. Am J Psychol.

[B21] Decety J, Grezes J (1999). Neural mechanisms subserving the perception of human actions. Trends Cogn Sci.

[B22] Jacobson E (1931). Electrical measurements of neuromuscular states during mental activities; 5. Variation of specific muscles contracting during imagination. Am J Physiol.

[B23] Kuhtz-Buschbeck JP, Mahnkopf C, Holzknecht C, Siebner H, Ulmer S, Jansen O (2003). Effector-independent representations of simple and complex imagined finger movements: a combined fMRI and TMS study. Eur J Neurosci.

[B24] Abbruzzese G, Assini A, Buccolieri A, Marchese R, Trompetto C (1999). Changes of introcortical inhibition during motor imagery in human subjects. Neuroscience Letters.

[B25] Naito E, Kochiyama T, Kitada R, Nakamura S, Matsumura M, Yonekura Y, Sadato N (2002). Internally simulated movement sensations during motor imagery activate cortical motor areas and the cerebellum. J Neurosci.

[B26] Weiss T, Hansen E, Beyer L, Conradi ML, Merten F, Nichelmann C, Rost R, Zippel C (1994). Activation processes during mental practice in stroke patients. Int J Psychophysiol.

[B27] Shackell EM, Standing LG (2007). Mind Over Matter: Mental Training Increases Physical Strength. N Am J Psychol.

[B28] Chainay H, Krainik A, Tanguy ML, Gerardin E, Le Bihan D, Lehericy S (2004). Foot, face and hand representation in the human supplementary motor area. Neuroreport.

[B29] Szameitat AJ, Shen S, Sterr A (2007). Motor imagery of complex everyday movements. An fMRI study. Neuroimage.

[B30] Page SJ, Levine P, Leonard A (2007). Mental practice in chronic stroke - Results of a randomized, placebo-controlled trial. Stroke.

[B31] Isaac AR (1992). Mental practice: Does it work in the field?. SO: Sport-Psychologist.

[B32] Verbunt JA, Seelen HA, Ramos FPR, Michielsen BH, Wetzelaer WL, Moennekens M (2008). Mental practice-based rehabilitation training to improve arm function and daily activity performance in stroke patients: a randomized clinical trial. BMC Neurology.

[B33] Braun SM, Beurskens AJ, van Kroonenburgh SM, Demarteau J, Schols JM, Wade DT (2007). Effects of mental practice embedded in daily therapy compared to therapy as usual in adult stroke patients in Dutch nursing homes: design of a randomised controlled trial. BMC Neurology 2007.

[B34] Holmes PS, Collins DJ (2001). The PETTLEP approach to motor imagery: A functional equivalence model for sport psychologists. J Appl Sport Psychol.

[B35] Smith D, Wright C, Allsopp A, Westhead H (2007). It's All in the Mind: PETTLEP-Based Imagery and Sports Performance. J Appl Sport Psychol.

[B36] Wright CJ, Smith DK (2007). The Effect of a Short-term PETTLEP Imagery Intervention on a Cognitive Task. JIRSPA.

[B37] Driediger M, Hall C, Callow N (2006). Imagery use by injured athletes: a qualitative analysis. J Sports Sci.

[B38] MacIntyre TE, Moran AP (2007). A qualitative investigation of imagery use and meta-imagery processes among elite canoe-slalom competitors. Journal of Imagery Research in Sport and Physical Activity.

[B39] Munroe KJ, Giacobbi PR, Hall C, Weinberg R (2000). The four Ws of imagery use: Where, when, why, and what. Sport Psychol.

[B40] Gowland C, Stratford P, Ward M, Moreland J, Torresin W, Van Hullenaar S, Sanford J, Barreca S, Vanspall B, Plews N (1993). Measuring physical impairment and disability with the Chedoke-McMaster Stroke Assessment. Stroke.

[B41] Moreland J (1993). Theoretical basis of the Chedoke-McMaster Stroke Assessment. Physiother Can.

[B42] Finch E, Brooks D, Stratford PW, Mayo NE (2002). Physical Rehabilitation Outcome Measures - A Guide to Enhanced Clinical Decision Making.

[B43] Huijbregts MPJ, Gowland C, Gruber RA (2000). Measuring clinically-important change with the activity inventory of the chedoke mcmaster stroke assessment. Physiotherapy Canada.

[B44] Valach L, Signer S, Hartmeier A, Hofer K, Steck GC (2003). Chedoke-McMaster stroke assessment and modified Barthel Index self-assessment in patients with vascular brain damage. Int J Rehabil Res.

[B45] Gowland C, VanHullenaar S, Torresin W (1995). Chedoke-McMaster Stroke Assessment: development, validation, and administration manual.

[B46] Bergland A, Laake K (2005). Concurrent and predictive validity of "getting up from lying on the floor". Aging Clin Exp Res.

[B47] Landau JD, Leynes PA, Libkuman TM (2001). Mental simulation increases physical performance estimates but not physical performance. SO: Journal-of-Mental-Imagery.

[B48] Schädler S, Kool J, Lüthi H, Marks D, Oesch P, Pfeffer A, Wirz M (2006). Assessments in der Neurorehabilitation.

[B49] Marolf M, Vaney C, Prosiegel M, König N (1996). Evaluation of disability in multiple sclerosis patients: a comparative study of the functional independence measure, the extended barthel index and the expanded disability status scale. Clin Rehabil.

[B50] Prosiegel M, Böttger S, Schenk T, König N, Marolf M, Vaney C, Garner C, Yassouridis A (1996). Der Erweiterte Barthel-Index (EBI) - eine neue Skala zur Erfassung von Fähigkeitsstörungen bei neurologischen Patienten. Neurol Rehabil.

[B51] Weimar C, Kurth T, Kraywinkel K, Wagner M, Busse O, Haberl RL, Diener HC (2002). Assessment of functioning and disability after ischemic stroke. Stroke.

[B52] Jansa J, Pogacnik T, Gompertz P (2004). An evaluation of the Extended Barthel Index with acute ischemic stroke patients. Neurorehabil Neural Repair.

[B53] Berg KO, Wood-Dauphinee SL, Williams JI, Maki B (1992). Measuring balance in the elderly: validation of an instrument. Can J Public Health.

[B54] Berg K, Wood-Dauphinee S, Williams JI (1995). The Balance Scale: reliability assessment with elderly residents and patients with an acute stroke. Scandinavian journal of rehabilitation medicine.

[B55] Stevenson TJ (2001). Detecting change in patients with stroke using the Berg Balance Scale. Aust J Physiother.

[B56] Conradsson M, Lundin-Olsson L, Lindelof N, Littbrand H, Malmqvist L, Gustafson Y, Rosendahl E (2007). Berg Balance Scale: Intrarater Test-Retest Reliability Among Older People Dependent in Activities of Daily Living and Living in Residential Care Facilities. PHYS THER.

[B57] Scherfer E, Bohls C, Freiberger E, Heise KF, Hogan D (2006). Berg-Balance-Scale - German Version - Translation of a Standardized Instrument for the Assessment of Balance and Risk of Falling. physioscience.

[B58] Hall C-R, Martin K-A (1997). Measuring movement imagery abilities: A revision of the Movement Imagery Questionnaire. Journal of Mental Imagery.

[B59] Malouin F, Richards C, Jackson P, Lafleur M, Durand A, Doyon J (2007). The Kinesthetic and Visual Imagery Questionnaire (KVIQ) for Assessing Motor Imagery in Persons with Physical Disabilities: A Reliability and Construct Validity Study. J Neurol Phys Ther.

[B60] Fournier JF (2000). IMAGIX: Multimedia Software For Evaluating the Vividness of Movement-Imagery. Percep Motor Skills.

[B61] Fournier JF, Deremaux S, Bernier M (2008). Content, characteristics and function of mental images. Psychology of Sport and Exercise.

[B62] Beaton DE, Bombardier C, Guillemin F, Ferraz MB (2000). Guidelines for the process of cross-cultural adaptation of self-report measures. Spine.

[B63] Malouin F, Belleville S, Richards CL, Desrosiers J, Doyon J (2004). Working memory and mental practice outcomes after stroke. Arch Phys Med Rehabil.

[B64] Bandura A (1977). Self-efficacy: toward a unifying theory of behavioral change. Psychol Rev.

[B65] Powell LE, Myers AM (1995). The Activities-specific Balance Confidence (ABC) Scale. J Gerontol A Biol Sci Med Sci.

[B66] Schott N (2008). German adaptation of the "Activities-specific Balance Confidence (ABC) scale" for the assessment of falls-related self-efficacy. Z Gerontol Geriatr.

[B67] Martin KA, Hall CR (1995). Using mental imagery to enhance intrinsic motivation. J Sport Exercise Psy.

[B68] Crosbie JH, McDonough SM, Gilmore DH, Wiggam MI (2004). The adjunctive role of mental practice in the rehabilitation of the upper limb after hemiplegic stroke: a pilot study. Clin Rehabil.

[B69] Kornspan AS, Overby L, Lerner BS (2004). Analysis and performance ofpre-performance imagery and other strategies on a golf putting task. Journal of Mental Imagery.

[B70] Folstein MF, Folstein SE, McHugh PR (1975). "Mini-mental state". A practical method for grading the cognitive state of patients for the clinician. Journal of psychiatric research.

[B71] Oldfield RC (1971). The assessment and analysis of handedness: the Edinburgh inventory. Neuropsychologia.

[B72] Shapiro DA (1981). Comparative credibility of treatment rationales: three tests of expectancy theory. Br J Clin Psychol.

[B73] Maher CG, Sherrington C, Herbert RD, Moseley AM, Elkins M (2003). Reliability of the PEDro scale for rating quality of randomized controlled trials. Phys Ther.

[B74] Lord SR, Shenington C, Menz HB (2001). Falls in older people. Risk factors and strategies for prevention.

[B75] Friedman SM, Munoz B, West SK, Rubin GS, Fried LP (2002). Falls and fear of falling: which comes first? A longitudinal prediction model suggests strategies for primary and secondary prevention. J Am Geriatr Soc.

[B76] Jorgensen L, Engstad T, Jacobsen BK (2002). Higher incidence of falls in long-term stroke survivors than in population controls: depressive symptoms predict falls after stroke. Stroke; a journal of cerebral circulation.

[B77] Pollock A, Baer G, Langhorne P, Pomeroy V (2007). Physiotherapy treatment approaches for the recovery of postural control and lower limb function following stroke: a systematic review. Clinical Rehabilitation.

[B78] Page SJ, Levine P, Leonard AC (2005). Effects of mental practice on affected limb use and function in chronic stroke. Arch Phys Med Rehabil.

[B79] Page SJ, Levine P, Sisto S, Johnston MV (2001). A randomized efficacy and feasibility study of imagery in acute stroke. Clin Rehabil.

[B80] Page SJ (2000). Imagery improves upper extremity motor function in chronic stroke patients: a pilot study. Occupational Therapy Journal of Research.

[B81] Reiser M (2005). Strength gains by motor imagery of maximal muscle contractions. Zeitschrift Fur Sportpsychologie.

[B82] Williams JG, Odley JL, Callaghan M (2004). Motor imagery boosts proprioceptive neuromuscular facilitation in the attainment and retention of range-of-motion at the hip joint. Journal of Sports Science & Medicine.

[B83] Corbin CB (1967). Effects of mental practice on skill development after controlled practice. Res Q.

[B84] Altman DG, Schulz KF, Moher D, Egger M, Davidoff F, Elbourne D, Gotzsche PC, Lang T (2001). The revised CONSORT statement for reporting randomized trials: explanation and elaboration. Annals of internal medicine.

[B85] dr dresing, pehl G f4audio 3.0.3 for Windows. Marburg; 2008:Solutions for digital recording & transcription.

[B86] Kvale S (2007). Doing interviews (Book 2 of The SAGE Qualitative Research Kit).

[B87] Bortz J, Döring N (2006). Forschungsmethoden und Evaluation: für Human- und Sozialwissenschaftler 4, überarb Auflage edition.

[B88] QSR international. http://www.qsrinternational.com/default.aspx.

[B89] Gibbs GR (2007). Analysing Qualitative Data (Book 6 of The SAGE Qualitative Research Kit).

[B90] Tremblay F, Léonard G, Tremblay L (2008). Corticomotor facilitation associated with observation and imagery of hand actions is impaired in Parkinson's disease. Experimental Brain Research.

[B91] Stanton BR, Williams VC, Leigh PN, Williams SCR, Blain CRV, Giampietro VP, Simmons A (2007). Cortical activation during motor imagery is reduced in Amyotrophic Lateral Sclerosis. Brain Research.

[B92] Liu KP, Chan CC, Lee TM, Hui-Chan CW (2004). Mental imagery for relearning of people after brain injury. Brain Inj.

[B93] Tamir R, Dickstein R, Huberman M (2007). Integration of Motor Imagery and Physical Practice in Group Treatment Applied to Subjects With Parkinson's Disease. Neurorehabil Neural Repair.

[B94] Fell NT (2000). Mental imagery and mental practice for an individual with multiple sclerosis and balance dysfunction. Phys Ther Case Rep.

[B95] Lejeune M, Decker C, Sanchez X (1994). Mental rehearsal in table tennis performance. Percep motor skills.

